# Mechanical Pressure Driving Proteoglycan Expression in Mammographic Density: a Self-perpetuating Cycle?

**DOI:** 10.1007/s10911-021-09494-3

**Published:** 2021-08-27

**Authors:** Gina Reye, Xuan Huang, Larisa M. Haupt, Ryan J. Murphy, Jason J. Northey, Erik W. Thompson, Konstantin I. Momot, Honor J. Hugo

**Affiliations:** 1 School of Biomedical Sciences, Gardens Point, Queensland University of Technology (QUT), Kelvin Grove, QLD 4059 Australia; 2 Translational Research Institute, Woolloongabba, QLD Australia; 3 Centre for Genomics and Personalised Health, Genomics Research Centre, School of Biomedical Sciences, Faculty of Health, Institute of Health and Biomedical Innovation, Queensland University of Technology (QUT), 60 Musk Ave, Kelvin Grove, QLD 4059 Australia; 4 School of Mathematical Sciences, Gardens Point, Queensland University of Technology (QUT), Kelvin Grove, QLD Australia; 5 Department of Bioengineering, University of Pennsylvania, Philadelphia, PA 19104 USA; 6 School of Chemistry and Physics, Queensland University of Technology (QUT), Brisbane, QLD Australia

**Keywords:** Mammographic density, Proteoglycans, Mechanical stiffness, Collagen, Carcinogenesis

## Abstract

Regions of high mammographic density (MD) in the breast are characterised by a proteoglycan (PG)-rich fibrous stroma, where PGs mediate aligned collagen fibrils to control tissue stiffness and hence the response to mechanical forces. Literature is accumulating to support the notion that mechanical stiffness may drive PG synthesis in the breast contributing to MD. We review emerging patterns in MD and other biological settings, of a positive feedback cycle of force promoting PG synthesis, such as in articular cartilage, due to increased pressure on weight bearing joints. Furthermore, we present evidence to suggest a pro-tumorigenic effect of increased mechanical force on epithelial cells in contexts where PG-mediated, aligned collagen fibrous tissue abounds, with implications for breast cancer development attributable to high MD. Finally, we summarise means through which this positive feedback mechanism of PG synthesis may be intercepted to reduce mechanical force within tissues and thus reduce disease burden.

## Introduction

Micromechanics of the extracellular environment plays an important role in carcinogenesis and cancer proliferation. The initial recognition of the differences between normal and cancer cell interactions with their micromechanical environments dates back to the mid-1970s [1]. In the late 1990s—early 2000s it was demonstrated that "sensing" by healthy cells of the mechanical properties of their environment affected the life cycle of the cell [2–4]. It is now well-recognised that tissue mechanical properties have important implications for oncogenesis and proliferation of cancers in general [5], and breast cancers in particular [6].

In this review we discuss the various mechanical forces within the body, with a focus on the molecular composition of the breast and how this potentiates mechanical force in association with mammographic density (MD). Mechanical forces in tissues are important not only in injury but also in healthy physiology, and we summarise the importance of mechanical force in health and development. We elaborate on a potential self-perpetuating cycle of mechanical force and extracellular matrix (ECM) creation within the context of MD. We review novel mathematical models, and how they can be used to predict cellular behaviour within these environments of varying stiffness. Focussing on the breast and the stiffness effects of MD, we define the potentiators of mechanical force within this context, the extracellular matrix proteoglycan proteins and how they function to exert force in the breast. We review how these molecules may be detected and how force may be molecularly determined. Delving deeper, we then review the effects of mechanical force within the cell itself, and then conclude with a review of existing and prospective therapies to reduce the cellular effect of mechanical forces within breast tissue, with a focus on reducing breast cancer risk associated with MD.

## Mechanical Properties of Soft Tissues: Empirical Overview

**Fig. 1:**
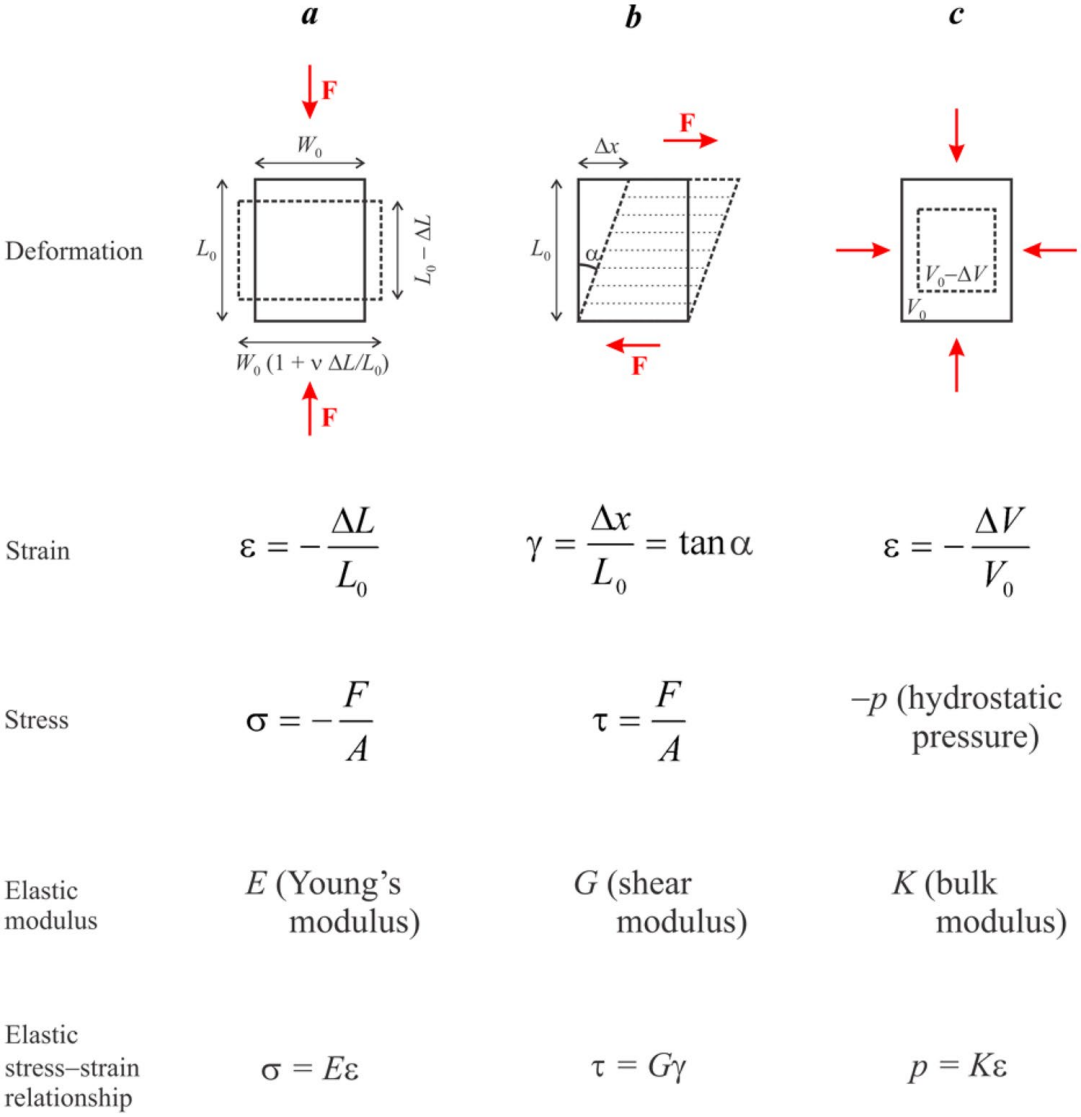
Stress, strain and elastic moduli for the three basic types of deformation: **a** Uniaxial compression; **b** Shear; **c** Volumetric deformation. Note that compressive strains and stresses are conventionally taken with the negative sign. In **a** and **b**, *A* is the cross-sectional area of the sample perpendicular to the direction of the force. In a, ν is the Poisson's ratio (see main text). For each type of deformation, strain is unitless, while stress and the respective elastic modulus have the units of Pa.

The relationship between cell function and the micromechanical environment of biological tissues is complex, and an understanding of these interactions and their impact on normal and pathological conditions ultimately requires consideration of the molecular machinery underpinning the physiology of the cell. However, the first step in untangling this complexity is information from the experimental characterisation of the *macroscopic* mechanical properties of the tissue. For the purposes of this review, we will limit the discussion to the three basic experimental settings illustrated in Fig. 1: uniaxial compression (or tension), Fig. 1a; shear, Fig. 1b; and volumetric (or hydrostatic) deformation, Fig. 1c.

Each scenario shown in Fig. 1 involves application of deforming forces and a measurement of the displacement of the tissue sample (mechanical response). However, the relationship between force and displacement does not of itself enable direct characterisation of the tissue properties: a given force, uniformly applied to a large sample, will cause a smaller deformation than the same force uniformly applied to a smaller sample. In order to enable characterisation of the tissue itself (rather than a specific sample of a given size), force is replaced with *stress*, σ = *F*/*A*, where *A* is the cross-sectional area of the sample perpendicular to the force. Similarly, displacement is replaced with *strain* (ε), defined as the displacement relative to the dimensions of the undeformed sample. Unlike force and displacement, strain and stress are independent of the size of the sample and characterise the tissue itself.

The specific definitions of strain and stress differ between the three types of deformation shown in Fig. 1, but their physical meanings are equivalent in each setup. This is reflected in their equivalent physical units. All three types of stress are measured in the units of force divided by cross-sectional area (SI units, Newton/m^2^ = Pascal [Pa], the physical unit of pressure). All three types of strain are unitless quantities. There are also equivalent stress − strain relationships describing each type of deformation, and each of the respective elastic moduli is measured in Pa (units of stress/strain).

In addition to the three elastic moduli, biological tissues are characterised by Poisson's ratio (ν). Its physical meaning is illustrated by Fig. 1a: while the sample is compressed in one direction, it expands in the orthogonal directions. This is a consequence of the incompressibility of water: without an outflow of water, unconstrained uniaxial compression results in a change of shape rather than a change in volume of the sample. Poisson's ratio characterises this change of shape in the limit ε → 0:

1 $$\nu =\left(\frac{{\Delta }W}{{W}_{0}}\right)/\left(\frac{{\Delta }L}{{L}_{0}}\right)$$

where the meaning of Δ*W*, *W*_0_, Δ*L* and *L*_0_ is illustrated in Fig. 1. For a completely incompressible, isotropic tissue, ν = 1/2. For a tissue that is able to undergo compression proper (e.g. through the outflow of tissue water), volume could in principle range from 0 and 0.5, but for most soft tissues it is close to 0.5.

The four parameters (Young's, shear, bulk moduli and Poisson's ratio; see Fig. 1) are mutually dependent. For an ideal elastic material, the knowledge of any two enables calculation of the others: *E* = 2*G*(1 + ν) = 3* K*(1 − 2ν). It should be noted that the values of the measured elastic moduli are generally dependent upon the spatial scale of the measurement: i.e., the mechanical properties of the tissue can differ between the macroscopic, mesoscopic and microscopic scales [7].

**Fig. 2:**
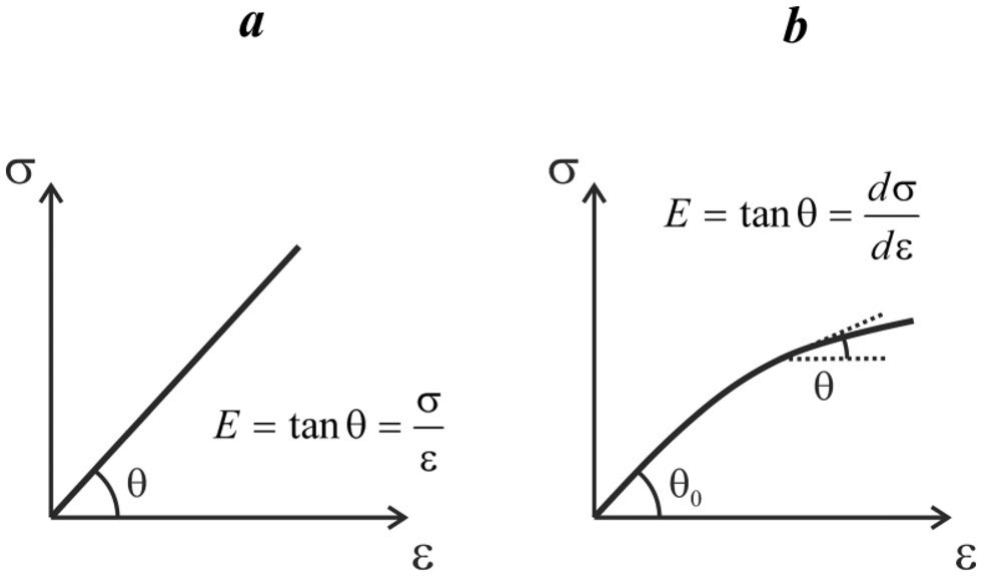
Elastic modulus for ideal and non-ideal materials: **a** For an ideal, purely elastic material the stress − strain relationship is linear as shown in Fig. 1. The elastic modulus is the slope of the stress-*vs*-strain plot; **b** Stress − strain relationship for a typical real biological soft tissue. The stress-*vs*-strain plot can be approximated as linear at small stresses (or strains), but the linear relationship is lost at larger stresses or strains. The elastic modulus can be defined as the instantaneous slope of the stress-*vs*-strain curve (this definition is known as the *tangent modulus*); such a modulus is stress- or strain-dependent. In **b**, the angle θ_0_ indicates the elastic modulus in the limit of small strain

The linear stress − strain relationships shown in Fig. 1 apply strictly only to an ideal material whose compressive response is purely elastic (an example is a hypothetical massless spring). The mechanical response of most real biological soft tissues is linear only in the limit of small stress (or small strain); at larger stresses or strains the linearity is lost. This is illustrated in Fig. 2. In general, the elastic modulus can be defined as the instantaneous slope of the stress-*vs*-strain curve. Unlike the ideal scenario illustrated in Fig. 1, such a modulus is no longer constant: it is stress- or strain-dependent and therefore represents a composite empirical parameter characterising the material under a given set of measurement conditions. Mechanical characterisation of the material in this case requires the sampling of the full stress − strain curve, as opposed to the measurement of the elastic modulus at a single value of stress or strain.

The modelling of the mechanical response of biological soft tissues commonly invokes the viscoelastic model, whereby the material possesses elasticity as well as viscosity [8, 9]. These two components of the viscoelastic response can be intuitively understood by considering the response to a periodic (oscillating) stress. The component of the response that is in sync with the input load is the elastic component, while the component lagging the input load is the viscous component of the response. Consequently, the elastic modulus is commonly represented as a complex quantity, with the real part (known as the storage modulus) representing the elastic response and the imaginary part (known as the loss modulus) representing the viscous response. The physical meaning of the two components is that the elastic response stores energy (which can be recovered when the deformation is reversed), while the viscous response irreversibly dissipates energy. The viscoelastic model is able to capture some important features of the dynamic mechanical response. These include strain creep (under constant stress, the strain rises gradually and plateaus) or stress relaxation (under constant strain, the stress is gradually dissipated), both of which are crucial to the understanding of many aspects of mechanical response of tissues.

## Experimental Characterisation of Mechanical Properties of Tissues

Mechanical testing of biological materials and tissues is a vast field of research, and its in-depth discussion is outside the scope of the present review. A detailed overview of the common testing methods and instrumentation can be found in the literature [10]. Broadly, experimental approaches to mechanical testing of biological tissues can be divided into two groups: (1) direct mechanical testing, which entails applying mechanical load(s) and directly measuring the appropriate stress or strain response; and (2) indirect characterisation of mechanical properties, which is usually based on some form of quantitative imaging. A very limited list of selected examples of both types of approaches can be found in Table 1.

**Table 1:** A selection of experimental methods of characterisation of mechanical properties of biological tissues

Method	Quantity measured	Direct/Indirect
Compressive testing	*E*	Direct
Tensile testing	*E*	Direct
Indentation	*E*	Direct
Rheometry	*G*	Direct
Magnetic Resonance Imaging (MRI)	Various characteristics, depending on the method used	Indirect
Elastography	Typically *G* (both Storage and Loss); also other characteristics, depending on the method used	Indirect
Nanoindentation	*E* (µm scale)	Direct
Atomic Force Microscopy (AFM)	*E* (nm scale)	Direct

The three deformation modes illustrated in Fig. 1 provide the setting for the basic "direct" mechanical testing approaches: for example, compressive and tensile testing (which yield Young's modulus *E*) or quasistatic shear testing (shear modulus *G*). Another common approach is indentation, whereby the stress–strain curve is sampled using a small indenter, which can have various shapes. Indentation is often the preferred approach to mechanical testing in vivo, where a uniform plate-induced compression may not be possible. Besides these basic approaches, direct mechanical-testing approaches include more sophisticated techniques, such as multiaxial testing [11], consolidation measurements (e.g. time-dependent creep and stress-relaxation measurements) [12], harmonic loading for the measurement of storage and loss moduli, or rheometry.

Indirect mechanical characterisation methods are typically based on quantitative imaging measurements where the quantity measured serves as a proxy for some physical property of the tissue [13]. Magnetic Resonance Imaging (MRI) is a highly versatile imaging modality that can provide access to a range of compositional, microstructural and mechanical characteristics of the tissue measured. For example, in articular cartilage MRI-based diffusion-weighted and diffusion-tensor imaging can provide microscopic-level insights into the poroelastic biomechanics of articular cartilage [14]. Elastography is another modality widely used for the imaging of mechanical properties of tissues. Elastography can be described as a "quantitative, noninvasive palpation" [15]. A typical elastography measurement entails introducing mechanical vibrations into the tissue using an actuator or an acoustic source, followed by acquisition of "wave image", which is then transformed into a map of the appropriate mechanical modulus. The imaging component is typically based either on ultrasound ("Ultrasound elastography") or MRI ("Magnetic Resonance elastography"). Magnetic Resonance Elastography was first reported in 1995 [16] and has since become an established and increasingly important diagnostic imaging modality. This is due in part to its success in breast imaging applications, including the ability to identify breast tumours [17].

It should be noted that many methods of characterisation of mechanical properties of tissues can contain elements of both "direct" and "indirect" approaches, whereby a direct measurement of the mechanical response is accompanied by an imaging measurement that provides spatially resolved information (often on the microscopic scale) about the distribution of strains or deformations [18, 19]. The information obtained in this case from the imaging measurement complements that from the direct mechanical measurement, enabling a more detailed insight into the mechanical response of the tissue on the microstructural level. The distinction between "direct" and "indirect" mechanical measurements can therefore be somewhat diffuse.

Table 2 provides a sampling of representative mechanical properties of several biological tissues.

**Table 2:** Representative mechanical properties of biological tissues. Note that, for a given tissue type, different measurement methods can produce significantly different results, depending on the measurement conditions and the spatial scale of the measurement

Tissue	Animal – Condition	Method	Property	Value	Ref
Eye lens (non-cataractous, cortex)	Human – young (< 30yo)	Mechanical rotation with optical measurement	*E*	0.75 – 1 kPa	[20]
		Indentation	*G*	100 – 160 Pa	[20]
	Human – old (> 60yo)	Mechanical rotation, optical measurement	*E*	3 kPa	[20]
		Indentation	*G*	1.5 – 2.5 kPa	[20]
Skin—forehead	Human	Indentation	*E*	4 – 12 kPa	[20]
Skeletal muscle	Rat	Tension	*E*	100 kPa	[21]
	Mouse	AFM	*E*	12 kPa	[21]
Achilles tendon	Human	Tension	*E*	65 MPa	[20]
	Rabbit	Tension	*E*	180 − 350 MPa at maximum strain	[22]
	Rat	Tension	*E*	310 MPa	[21]
Articular cartilage	Bovine	Compression	*E*	950 kPa	[21]
	Rabbit—Femoral condyle	Indentation − whole condyle Creep test	*G*	300 − 600 kPa (unrelaxed) 60 kPa (relaxed)	[22]
	Human	Unconfined compression	*E*	8.4 – 15.3 MPa	[20]
		Various	ν	0.37 – 0.5	[20]
	Human—Tibial plateau	Confined compression	*E*	5.1 – 7.9 MPa	[20]
			*K*	31 – 56 MPa	[20]
		Torsional creep and stress relaxation	*G*	2.6 – 4.1 MPa	[20]
Cortical bone from femur	Human	Tension and compression	*E*	10 – 20 GPa (anisotropic)	[23]
			ν	0.46 – 0.58 (anisotropic)	[23]
		Torsional tests	*G*	3.3 GPa	[23]
Fat	Human	Indentation	*E*	17 Pa	[21]
		Compression	*E*	25 kPa	[13]
Mammary gland	Human	Compression	*E*	160 Pa	[21]
			*E*	45 − 60 kPa	[13]
Pre-malignant ductal carcinoma in situ (DCIS)	Human	Indentation	*E*	2.2 kPa	[21]
		Compression	*E*	50 – 150 kPa (strain-dependent)	[13]
Breast tumours	Human	Compression	*E*	4 kPa	[21]
			*E*	50 – 300 kPa (strain-dependent)	[13]

## Biophysical Basis of the Mechanical Properties of Breast Tissue

Human breast tissue has two major components: fibroglandular tissue (FGT) and adipose tissue (fat). FGT is comprised of two distinct tissue types: connective tissue stroma and epithelial tissue of the mammary gland. Stroma forms a soft “internal skeleton” supporting the lobules of the mammary gland and is comprised primarily of fibroblast cells and the extracellular matrix, with the epithelial cells of the mammary gland having secretory functions. Adipose tissue surrounds the FGT [24–26]. The FGT to fat ratio within breast tissue can vary significantly between adult individuals, for reasons yet unknown, however it is this ratio that is the main physical correlate of radiographically measured MD.

### Mechanical Properties of Fibroglandular Tissue

Stroma occupies the majority of the FGT volume in a non-pregnant, non-lactating breast. It is therefore the main determinant of the mechanical properties of FGT. It should be noted that the stroma itself is mechanically non-uniform as it is comprised of perilobular connective tissue that is relatively stiff, while intralobular connective tissue is relatively loose, allowing expansion of the mammary glands during pregnancy [24].

In the first approximation, the mechanical properties of the stroma are determined by its extracellular matrix (ECM). Physico-chemically, the ECM can be thought of as a hydrogel consisting of a hydrated mixture of collagen, glycosaminoglycans (GAGs) and proteoglycans (PGs). The main structural collagens of the FGT ECM are the fibrillar collagens, Type I and III collagens [27]. The "building block" of the fibres is the tropocollagen molecule, which has the geometry of a straight thin rod 300 nm in length and 1.5 nm in diameter [12, 28]. Tropocollagen molecules self-assemble, through a combination of covalent and electrostatic interactions, into fibres. The assembly has a 67 nm stagger, which gives collagen fibres their characteristic banding in electron micrographs and the Bragg reflections in small-angle X-ray scattering, along with a characteristic and predictable pattern in 2^nd^ harmonic generation microscopy [29, 30]. Collagen fibres form a cross-linked network and it is this network that gives the ECM its *tensile strength*; serving as a three-dimensional (3D)scaffold that anchors GAG and PG molecules, forming the structural core of the ECM and holding it together. The other significant structural collagen of the FGT stroma is non-fibrillar Type IV Collagen, which is a major component of basement membranes [26, 31]. Type IV Collagen performs a similar function to Type I and III collagens, namely the anchoring of PG molecules [28].

GAGs are large, linear polysaccharide molecules typically composed of repeating disaccharide blocks. A common GAG within FGT ECM is heparan sulphate, which is built of blocks of glucuronic acid and N-acetylglucosamine. The carboxyl and sulphate groups of GAGs are negatively charged, a property crucial to their ability to osmotically attract ECM water. PGs are macromolecules with a protein core to which branching GAG chains are covalently attached. On the basis of their localisation, PGs are categorised as ECM-secreted, cell surface-associated or intracellular. Secreted PGs include hyalectans (aggrecan, versican, brevican and neurocan), small leucine-rich PGs (SLRPs) (decorin, biglycan and lumican) and basement membrane PGs (perlecan, agrin, collagen 3 and 8). Heparan sulfate proteoglycans (HSPG) include two main cell surface subfamilies, syndecans and glypicans [32, 33], with serglycin found to be the only intracellular PG [33]. PGs can connect directly with collagen [34] or indirectly via integrin bridges [35], sequester growth factors [36], and can effect multiple biological events including proliferation, differentiation and gene expression [37, 38].

PG molecules have the molecular weight (MW) of ~ 10^6^ Daltons (Da), and their aggregates can have MW ~ 10^9^ Da. Such large molecules are effectively trapped by the collagen network, and the negatively charged carboxyl and sulphate groups form a cloud of fixed electric charge within the ECM. This fixed electric charge acts as an osmotic sponge, enabling polysaccharide molecules to retain large amounts of water in the ECM. This is a key determinant of *elasticity* of the ECM: incompressible water is osmotically trapped by the polysaccharide molecules, which means that the outflow of water under mechanical compression is limited and the tissue is able to resist compression [39]. The outflow of water can be further limited by the finite hydraulic permeability of the ECM biopolymer network (the poroviscoelastic model of tissue elasticity) [14].

### Mechanical Properties of Adipose Tissue

Unlike the FGT stroma, adipose tissue is a high-cellularity tissue. Adipocytes are tightly packed large cells (50–100 + μm) that form fat. Despite the large relative volume occupied by adipocytes, the mechanical properties of adipose tissue are primarily controlled by extracellular collagenous structures rather than the cells themselves. Each adipocyte is surrounded by a reinforced basement membrane of ~ 100 nm thickness. This Type IV collagen membrane is adjacent to the cell's phospholipid membrane. The basement membranes collectively form a 3D closed-shell foam with the relative density ~ 0.1 [40]. Adipose tissue also contains sparse interlobular septa, which are fibrous bundles consisting predominantly of Type I collagen. The septa are several mm long and 10 – 30 µm thick, the characteristic distance between septa bundles is ~ 1 mm, and they occupy the relative volume ~ 3⋅10^−4^. These two collagenous microstructures are the main determinants of the elastic properties of adipose tissue. The septa provide a negligible contribution to the elastic modulus due to the relatively small volume they occupy, and the main contribution to the elastic modulus comes from the 3D network of basement membranes. However, due to their preferential alignment, the septa have been postulated to determine the anisotropy of the mechanical properties of the tissue [41].

## Mammographic Density: Molecular Basis and Implications of Carcinogenesis

MD refers to the relative proportion of fibroglandular to adipose tissue in the breast, and MD has been implicated as an independent risk factor for breast cancer [42]. MD can be characterized as the relative amount of fibroglandular stroma containing ECM [43]. The preponderance of water-carrying PG molecules within this stroma is evidenced, among other research, by our pioneering work using single-sided portable nuclear magnetic resonance (NMR) as a technique for quantification of MD and its change in human tissue cultured ex vivo [44, 45]. Of particular interest is the proteoglycan versican, which accumulates in breast cancer stroma and has been reported to be correlated with high MD [46].

Numerous epidemiological investigations have consistently demonstrated that an increase in percent MD (PMD) is an independent risk factor of breast cancer and progression [47]. It is also known that on a population level, high MD-associated breast cancer risk is more prominent than other known risk factors, such as the BRCA1 and BRCA2 predisposition genes [48], with approximately 45% of women fall into Breast Imaging-Reporting and Data System (BIRADS) categories of C and D [49]. Patients with PMD values greater than 75% confer 4 to sixfold higher risk of breast cancer comparative to those with values lower than 10% [50]. In addition to breast cancer risk prediction, breast cancer patients in the high MD classification are also 16% more likely to acquire local disease recurrence [51], and are thus more susceptible to therapy resistance [52]. Research has shown that DCIS lesions occur primarily in areas of high MD, where it has been positively associated with lymph node status, tumour size and vascular or lymphatic invasion [53]. The accumulation of collagen has been shown to influence features of mammary malignancy in vitro [54] and in vivo [55], where aligned collagen in high MD is a predictor for poor survival in breast cancer [56].

## Mechanical Forces in Health

Mechanical forces are imperative in normal cellular development [57]. Branching morphogenesis is a phenomenon which is critical in embryonic development of several tissues, such as neural tissue, but also glandular epithelia in several organs such as the liver, pancreas, salivary glands, lung, and also the breast. The breadth and directionality of the specific branching depends on ECM composition and how it is organised [58]. Furthermore, mechanical force, in addition to cell–cell and cell–matrix attachment, along with soluble factors, dictate the type of cell that mesenchymal stem cells become after differentiation [59]. For example, stem cells cultured on substrates with stiffnesses similar to muscle become myogenic, while those cultured on substrates with stiffnesses similar to bone become osteogenic [60]. In regard to mammary gland development, insights into the role of mechanical force have been provided by the work of Provenzano and colleagues, who demonstrated that mammary epithelial cells cultured in soft matrices are proliferative and exhibit ductal expansion, while stiffer matrices promote an epithelial-to-mesenchymal transition enabling invasion and differentiation of the epithelial cells [61]. As such, mechanical interaction between cells and their surroundings during embryonic and adolescent pubertal development likely helps to shape the mammary ductal/lobular tree [61].

Mechanical forces are also important in the development and maintenance of many connective tissues. This was brought to the fore in the study of the effects of space flight on the human body, leading to cellular research in microgravity environments [62]. These mechanical unloading experiments revealed that gravity is necessary for optimal bone, cartilage and muscle density and strength [63], widely established for the beneficial effects of weight-bearing exercise on Earth. Specifically, in the articular cartilage of mice cultivated in the microgravity environment of space, *proteoglycan* content was reduced, an effect also observed in knee cartilage and intervertebral discs of immobile incapacitated individuals [64]. Mechanical loading is also instrumental to the development of aligned zonal structure of the collagen fibre network in articular cartilage [65]. Mechanical loading experiments performed on collagen gel samples in vitro have shown strain-induced alignment of collagen fibres [66]. This alignment was reversible in cross-linked collagen gels but irreversible in samples with no cross-linking, suggesting that the development of collagen network alignment in articular cartilage may involve an interplay of mechanical loading per se and inter-fibre molecular interactions or cross-linking. Conversely, repeated application of mechanical load is capable of irreversibly altering an existing collagen network alignment in collagenous connective tissues [67].

## Mathematical Models of Stiffness

Mathematical models are a valuable tool to help interpret biological experiments and provide a framework to develop mechanistic understanding otherwise difficult if not impossible with experimentation alone. While many mathematical models are utilised to explore cancer development [68], mammary branching morphogenesis [69–71], and mechanical pressure in tissue development [72], few mathematical models have to date connected mechanical pressure, proteoglycan expression, and MD. In the following we highlight some recent biologically motivated mathematical modelling studies in the field.

Cancer development has been studied widely with a variety of mathematical models and techniques. As discussed by others [73, 74], models of tumour development can broadly be classified as: (i) discrete, where individual cell properties and interactions are prescribed and stochastic effects can be included; or (ii) via continuum models, which consider the behaviour of continuous cell densities and chemical concentrations as opposed to the behaviour of individual cells; or (iii) using hybrid discrete-continuum models. Simmons et al. [75], with a particular focus on breast cancer development, invasion, and therapies, review discrete models, continuum models which typically take the form of a system of differential equations [76, 77], and multi-scale models where processes across multiple time and/or spatial scales are incorporated [78]. Furthermore, they consider a cellular Potts model (CPM), as an example of a lattice-based model where each cell is represented by a subset of lattice sites with the same cell composition and the simulation is updated by minimising the total energy in the system. While the CPM seems abstract there are many biological applications, for example Boghaert and colleagues [79] show that by varying the relative rates of key parameters such as contractility and proliferation four different ductal carcinoma in situ morphologies can emerge, consistent with clinical histological data. Image-based models provide an alternative approach and are comprised of three key steps: i) experimental image analysis and quantification, ii) modelling, and iii) simulation [75, 80]. As high MD may lead to metastatic progression facilitated by epithelial to mesenchymal transition (EMT), we highlight recent novel multi-organ metastatic growth models [81, 82]. Mathematical models with experimental validation also provide insights into the frequency and size distributions of circulating tumour cell clusters leaving a primary site and entering the metastatic cascade [83] along with molecular mechanisms that enable formation of clusters in highly aggressive inflammatory breast cancer [84].

As we have alluded to earlier, the ECM plays an important role in breast tissue development, stiffness as well as tumour initiation and growth through coupled mechanobiological/mechanochemical mechanisms [85–88]. First focusing on mechanical properties, ECM stiffness is important as cells feel and respond to the stiffness of their substrate [4]. This can lead to changes in cell movement, and the proliferative and metastatic potential of the cancer and stromal cells [89]. Movement in the direction of an increasing stiffness gradient is referred to as durotaxis [90]. Durtotaxis can be explored using a clutch model in which local stick–slip dynamics of cell–matrix adhesions are integrated at the tissue level through cell–cell junctions [91], a cellular Potts model extended to include the mechanical response of focal adhesions [92], and discrete models where the position of an individual cell updates at a rate dependent on the stiffness at its current position [93]. Furthermore, the role of ECM density and stiffness on cancer cell invasion [94–96] EMT [97, 98], and stress giving rise to proliferative disorders and avascular tumour growth can also be explored with models [99].

As the ECM influences cellular behaviour, changes in cellular properties influence the ECM resulting in a two-way feedback mechanism. Models of this two-way feedback between contractility and matrix realignment suggests a nonlinear mode of cancer cell invasion [100]. Furthermore, experimental results show that actomyosin-mediated cellular tension drives increased tissue stiffness and $\beta$-catenin activation to induce epidermal hyperplasia and tumour growth [101], and cell contraction induces long-range stress stiffening in the ECM [102]. The stiffness of tumour biopsies and single cells show unique fingerprints that identify the different stages of cancer [103, 104] which is promising for cancer detection. Mathematical models can be used to interpret and explore these experimental observations [95, 105, 106] but more work is required.

Along with mechanical properties, chemical signals and intracellular networks are important for tissue functionality and the ECM is a key regulator [107]. Furthermore, mechanochemical feedback loops are thought to play an important role in development and disease both at the molecular level via mechanosensation, and at the cellular and tissue level [108]. Recent experiments [109] in epithelial tissues and related mathematical modelling [109] explore spatio-temporal waves of density and ERK/MAPK activation in an effort to characterise this regulation. The coupling of the Rac-Rho pathway or YAP/TAZ signalling with mechanical tension can be captured in mathematical models [110–112]. More relevant to MD, mathematical models have been used to explore the role of proteoglycan expression. Magzoub et al. [113] combine experimental work with mathematical modelling to determine the role of ECM components in macromolecule diffusion deep in the tumour with applications to drug delivery. They quantify the roles of extracellular space volume fraction to indicate a substantial effect of cell density on diffusion in deep tumour and experimentally find macromolecule diffusion is enhanced deep into the tumour after enzymatic digestion of ECM collagen and its associated proteoglycan decorin. As proteoglycans trapped in the ECM generate high levels of osmotic pressure to counter-balance external pressures, Lu et al. [114] use a mathematical model to find that the viscoelastic behaviour of soft tissues significantly depends on the contribution of osmotic pressure in the ECM during deformation. Other models focus on ECM remodelling with different techniques including: a poroelastic model where tumour mechanical resistance is primarily attributed to GAG swelling [115]; a porous solid matrix with Green-elastic and elasto-visco-plastic material behaviour [116] and lattice models describing ECM fibre degradation, realignment and deposition [117]. While other approaches model the ECM as a continuum, with epithelial cells modelled as individual cells to explore how cross-talk between stromal and tumour cells influences biochemical and mechanical properties and resulting tumour evolution [118].

## Intracellular Effects of Mechanical Force

### The Force Awakens: Integrins, Where Mechanosensing Begins

One of the first extracellular sensors of mechanical force are the integrins, transmembrane proteins and heterodimeric receptors which exist in 24 unique combinations of non-covalently linked α-subunits (18 forms) and β-subunits (8 forms) [119]. Of relevance to mechanical sensing, integrins relay signals between intracellular and extracellular pathways to facilitate binding to a wide array of ECM components via focal adhesions [120]. Whilst mediating cell adhesion via these focal contacts, integrins are responsible for the transmission of signals across the plasma membrane and via actin to regulate cell survival, growth and migration [121]. Integrin activation increases the binding of integrin extracellular domains to ligands, which encompasses both changes in affinity of individual integrins and increased clustering due to conformational changes [122, 123]. Whilst expression is typically regulated by mRNA expression, integrins are continuously endocytosed and recycled to the plasma membrane, mediating the normal ratios of receptors between the cell surface and endosomal pools [124, 125]. The secretion of fibronectin, activation of cytoskeletal contractility and activation of focal adhesion kinase (FAK) have all been associated with an integrin-induced emergence of tumorigenic cells from dormancy [126]. The linkage between the cytoskeleton and integrins involves a multitude of complex integrin-associated proteins, which function in both the assembly and disassembly of the association [127]. Most integrins attach to the actin cytoskeleton via the integrin cytoplasmic tail and integrin-actin bridging proteins,, however the specialised integrin α6β4 connects with intermediate filaments to form hemidesmosomes [128]. Integrin-associated proteins such as parvin, talin, filamin, tensin and α-actinin contain actin-binding domains and are crucial for cellular adhesion to the actin cytoskeleton [129]. The integrin connection to actin is mediated by three mechanisms; nucleation of new filaments, capture of actin filaments and inhibition of specific actin structures [130]. Focal adhesions comprise large macromolecular attachment sites where regulatory signals are transmitted between the ECM and interacting cells [131]. Furthermore, focal adhesions can link cytoskeletal networks directly to the ECM, enabling cells to respond to the external environment. These structures also function to assemble and regulate multiple signalling pathways, typically activated when cells are attached to stiffer substrates [94]. The extracellular subdomains located on integrin subunits recognise ECM proteins and other receptors, where their cytoplasmic b-tails habitually interact with actin via talin and thus the cytoskeleton-signalling network with focal adhesion proteins such as tyrosine kinase Src, FAK, vasodilator-stimulated phosphoprotein (VASP) paxillin and integrin-linked kinase [132–134]. Mechanical strain induces FAK activation in multiple cell types which results in increased cellular proliferation through the activation of ERK/MAPK via multiple signalling pathways [135, 136]. Cellular migration can be regulated by FAK by acting as a scaffold for phosphorylation of Src and regulation of the RhoA-Rho-associated protein kinase (ROCK) pathway [137, 138].

### The Hippo signalling pathway, Yes-associated protein 1/ WW-domain-containing transcription regulator 1 (YAP/TAZ) and TEA domain family member (TEAD): converting mechanical force to transcriptional outputs

YAP1 and TAZ are transcriptional coactivators which transmit signals between the nucleus and cytoplasm and are encoded by paralogous genes [139]. Both YAP and TAZ are implicated within the Salvador-Warts-Hippo (SWH) or Hippo-pathway, where Hippo signalling exerts a critical role in modulating cellular homeostasis [140]. The Hippo signalling pathway is also composed of large tumour suppressor 1 and 2 (LATS1/2) and mammalian Ste20-like kinases 1 and 2 (MST1/2), and both inhibit the downstream activity of YAP/TAZ [141]. Mechanical forces including stress, strain, stiffness of ECM and cellular distortion are known to regulate both the localisation and activity of YAP/TAZ, dependent on the activity of Rho GTPases [142, 143]. Multiple studies have demonstrated that many extracellular ligands and growth factors regulate the Hippo pathway [144, 145]. EGF, through EGF-receptor (EGFR), inactivates the Hippo pathway by activation of PI3K and phosphoinositide-dependent kinase (PDK1) [146]. Alternatively, EGFR activates the YAP/TAZ pathway through the MAPK signalling axis [147]. YAP/TAZ translocate directly to the nucleus and interact with TEAD transcription factors 1–4 [148]. TEAD transcriptional networks are comprised of genes involved in cell proliferation, growth, and tissue homeostasis [149].

The Ras association domain family protein1 isoform A (RASSF1A) is a well-studied tumour suppressor protein and an upstream component of the Hippo pathway, responsible for regulation of proliferation, cell survival and mechanotransduction [150, 151]. RASSF1A binds to MST1/2 kinases and adaptor protein WW45 (SAV1) via the SARAH motif [152], where such interaction enables the regulation of apoptosis in response to DNA damage, EMT, autophagy initiation and elevations in tissue stiffness [153, 154]. The pro-apoptotic action of RASSF1A is mediated through direct interaction with Hippo kinases MST1/2, which prevents their inactivation and dephosphorylation [155, 156]. Prolyl 4-hydroxylase subunit alpha 2 (P4HA2) is an enzyme which catalyses the formation of 4-hydroxyproline for the biosynthesis of collagen [157]. The Hippo pathway mediator YAP1 regulates P4HA2 levels, which in turn has been documented to be tightly regulated by RASSF1A [158].

## Chromatin Re-organisation Triggered by Mechanical Forces on the Cell

Chromatin organisation comprises one of the major chromatin-remodelling events that occurs during mitosis, where alterations in chromatin compaction are vital in ensuring precise transmission of the replicated genome to daughter cells [159]. Lamin A/C (LMNA) is an inner-nuclear membrane protein involved in DNA repair, chromatin organisation and DNA repair [160, 161]. LMNA interacts with a multitude of large chromatin domains, referred to as lamin-associated domains (LADs), which have known association with eukaryotic cell differentiation [162]. It has been established that LMNA protein abundance and localisation at the nuclear envelope and within the nucleoplasm increases with tissue stiffness, alongside levels of collagen within the ECM [65]. A study has shown an increase in LMNA localisation and abundance within tumours comparative to healthy tissues, correlating with a stiffened microenvironment [163]. Direct support of a connection between mechanical force promoting ECM production, as may occur in MD, can be gleaned from study of Hutchinson-Gilford Progeria Syndrome (HGPS). HGPS is a disease characterized by reduced ECM protein synthesis, and individuals age rapidly as a result. In normal cells, LMNA is localised both at the nuclear envelope but also within the nucleoplasm. In HGPS LMNA is only found at the nuclear envelope, meaning that it cannot interact with specific lamin A—binding proteins to bind to euchromatin to promote synthesis of ECM proteins [164]. Taken together, these findings suggest that LMNA as a key potentiator of mechanical force and translation of this force into ECM production.

## Long-term Mechanical Force Promoting Tumour Suppressor Gene Silencing via Methylation

RASSF1A inactivation is a common molecular change observed in cancers [165]. In addition, hypermethylation of the RASSF1A promoter CpG island silences gene expression in multiple cancers, including prostate, glioma, lung, breast, and neuroblastoma [166]. Studies have demonstrated that the silencing of RASSF1A is associated with YAP1-driven activation of P4HA2, indicative that high collagen deposition and elevation in tissue stiffness results in RASSF1A deactivation [167, 168]. Indeed, increased tissue microenvironmental stiffness has been shown to induce DNA pro-methylation gene, DNA Methyltransferase 1 (DNMT1) [169]. It has also been shown that tumour cells containing activated YAP/TAZ display resistance to chemotherapy [170]. The deregulation of Hippo signalling and subsequent activation of YAP/TAZ is thought to directly activate the MAPK pathway, which is also frequently mutated in cancer [171]. Interestingly, we find RASSF1A gene and protein expression to be modulated by synthetically generated stiffnesses relevant to MD, suggesting that early RASSF1A gene expression changes may associate with increased breast cancer risk [172].

## Mechanical Force Driving PG Production: A Self-perpetuating Cycle?

Just as mechanical force appears to promote proteoglycan content and collagen alignment in joints in the body, mechanical force potentiated by increased fibroglandular stroma in MD states may participate in a self-perpetuating cycle. Indeed, high MD has been associated with increased stiffness and thus force on the epithelial cells [6].

Akin to the effect of mechanical force on cartilage, we find that proteoglycans are more abundant in stiffer, high MD human tissues compared with low MD tissues. In a recent study, proteomic profiling of non-malignant human breast samples revealed elevated levels of SLRPs, such as Biglycan (BGN) and Lumican, in mammographically dense tissue [45]. There is evidence that high levels of BGN are associated with poor prognosis in several cancers [173, 174], with conflicting results suggesting it may also hinder tumour cell growth [175]. BGN promotes FAK activation as seen by increased phosphorylation and gastric cancer invasion indicating that it can enhance cell mechanotransduction [176]. In breast cancers, BGN was found to play a role in establishing a breast cancer supportive ECM environment [177], and a separate study suggested that high BGN levels were associated with immune-mediated suppression of tumour progression and better prognosis [178]. This is in agreement with data that BGN can induce proinflammatory signalling through toll-like receptors in macrophages [179]. Context is surely critical, as BGN expression was induced in TGFβ treated cancer-associated fibroblasts (CAFs), implying involvement in generating a fibrotic stroma and an invasion permissive environment [180]. BGN may also promote tumor growth and chemotherapeutic resistance through the activation of NFκB signalling to maintain stem-like tumor cells as was shown in colon cancer [181]. While still relatively undefined, these data suggest that BGN may promote cancer progression and that its expression is favoured by elevated mechanical cues. Stromal expression of Lumican in breast cancer is associated with high tumor grade, low estrogen receptor expression and young age [182, 183]. Similarly, Lumican expression was found to be more prevalent in high grade pancreatic cancers and cytoplasmic levels were elevated in advanced colorectal cancer [184, 185]. The fact that Lumican levels correlate with MD suggest that it contributes to cancer risk and a permissive environment for breast cancer progression [45, 186].

Collagen XII, a Fibril-Associated Collagens with Interrupted Triple helix (FACIT) type collagen, was also determined to be significantly more abundant in MD breast tissues [45]. Consistently, Collagen XII was identified in independent proteomic screens to be associated with an inflammation-related stroma in breast cancer and a myofibroblastic signature enriched in regions of colon cancer invasion [187, 188]. Suggestive of a general role in promoting tumor progression, collagen XII was also found to be prognostic of overall survival in Pancreatic ductal adenocarcinoma [189].

Collagen XII assembles as a homotrimer with an N-terminal NC3 non-collagenous domain having three flexible arms with GAG side chains [190]. A C-terminal collagen helix links collagen XII to collagen-I fibres, decorin and fibromodulin, while the NC3 domain links it to other ECM proteins, such as Tenascin-X [190]. In this way, collagen XII is thought to organize collagen fibre bundles and regulate the mechanical properties of the ECM, particularly in more dense connective tissues and bone [190, 191]. Mutations in collagen XII are associated with connective tissue pathologies such as Ehlers-Danlos/myopathy overlap syndrome in humans, and mouse models display skeletal abnormalities and muscle weakness [190, 192]. For example, *Col12A1* knockout mice display defects in osteoblast and osteoclast function and disorganized collagen meshworks in bone [190, 193, 194]. Notably, mechanical strain induces collagen XII expression in a mouse osteoblastic cell line [195]. *Col12a1* deficiency further alters tenocyte shape, the formation of interacting cell processes, and organization resulting in impaired cell–cell communication with disruption of hierarchal structure and decreased tissue stiffness in tendons [196]. In skin, loss of collagen XII results in aberrant collagen network suprastructure and delayed wound healing due to an inability to sequester TGFβ and activate myofibroblasts [197].

Tensile stress results in increased production of collagen XII. For example, fibroblasts cultured in stretched collagen gels expressed higher levels of collagen XII, reduced upon relaxation of gel tension [198]. Chronic muscle loading and tooth movement result in induction of collagen XII in muscle and periodontal ligaments respectively [192, 199, 200]. Interestingly, two enhancer regions that control collagen XII induction and respond to either tensile or cyclic strain have been identified [195, 201]. The latter was discovered in mouse osteoblasts with known binding sties for c-Jun and JunD, while the former was identified in chicks with uncharacterized transcription factor binding sites [195, 201]. Overall, these data support the necessary integration of several mechanical cues for regulating collagen XII expression which can in turn modify the physical properties of the ECM, presumably in an effort to maintain tensional homeostasis. Collagen XII levels have also now been identified as a biomarker linked to fibrosis and breast cancer progression [45, 187, 202, 203]. Interestingly, in addition to elevated expression in tissues with high MD, we find collagen XII and XVI expression to be increased in human patient derived breast cancers grown within a stiff ECM environment in vivo (JJN unpublished observations). These data are supportive of a role for FACIT collagens in coordinating mechanosensitive responses as cells attempt to regulate and restore their mechanical integrity.

The heparan sulfate proteoglycan Syndecan 1 (SDC1) is more abundant in breast cancer [45, 204]. In normal breast tissue within the context of MD, similar to stiffened, cartilaginous collagen under pressure, collagen in high MD adjacent to glands is more aligned [205]. SDC1 interacts with collagen: SDC1 has been shown to act as a key co-receptor for $\alpha$2$\beta$1 integrin to enable adhesion to fibrillary type I collagen [206], and SDC1 expressed on the cell surface has been shown to maintain aligned collagen via an αvβ3 integrin bridge [35]. By thwarting the effect of SDC1 in maintaining aligned collagen via the αvβ3 bridge, we have shown that this alignment is necessary in mediating MD in an ex vivo model [45].

**Fig. 3:**
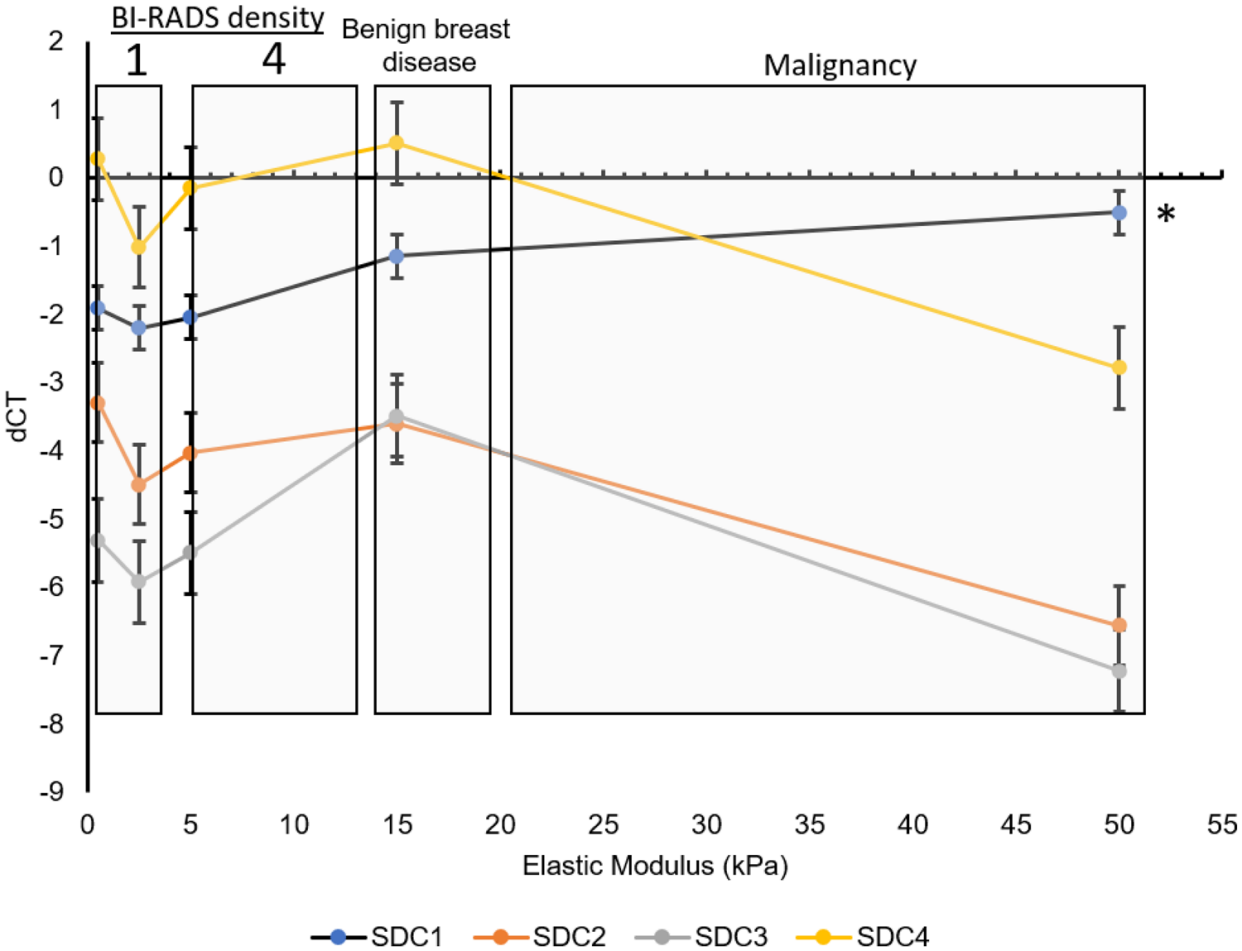
Expression of Syndecan genes 1–4 in MCF10DCIS.com cells cultured in 3D extracellular matrix mimic – GelMA (Gelomics) tuned to stiffnesses relevant to MD (BiRADs density is indicated), benign breast disease and stiffnesses found in malignant breast tumours

We have also performed experiments examining the 3D effect of increasing stiffness on epithelial cells and find that stiffness and the associated mechanical forces alone can specifically lead to increased SDC1 expression, which was significantly upregulated in MCF10DCIS.com cells cultured in increasingly stiff 3D matrices, ranging from 0.5 – 50 kPa (Fig. 3). Stiffness ranges relevant to the Volpara BIRADs density cut offs (BIRADs 1 = 0–4.5%, BIRADs 2 = 4.5–7.5%, BIRADs 3 = 7.5–15.5%, BIRADs 4 > 15.5%) were calculated from Fig. 1 data and overlaid according to stiffness values, on Fig. 3, to illustrate gene expression changes relative to biologically known stiffness ranges.

**Fig. 4:**
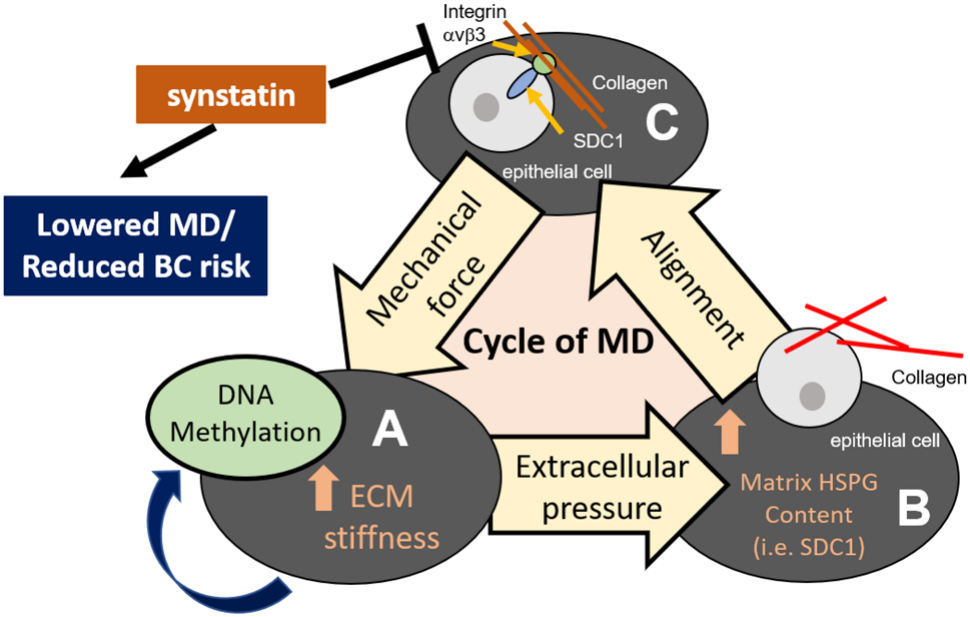
Summary schematic linking stiffness, proteoglycan expression and aligned collagen as a perpetual cycle promoting MD. **a** MD creates a microenvironment of increased stiffness via an abundance of ECM, which has parallels to cancer-activated stroma. This stiff environment of MD can promote tumour suppressor gene silencing by methylation, thus providing a direct link to breast cancer development due to high MD. **b** Stiffness creates increased extracellular pressure, promoting HSPG formation, the focus of this review. **c** SDC1 physically aligns collagen into stiffer, parallel fibres, via an integrin $\alpha$v$\beta$3 bridge. Synstatin thwarts the SDC1-integrin interaction with collagen, reducing collagen alignment and hence MD, as shown in our patient derived explant model of MD change

Given the role of SDC1 in maintaining MD, our data suggests a positive feedback cycle at play, where mechanical force itself may maintain proteoglycan (SDC1) expression to facilitate collagen alignment, and thus stiffness (Fig. 4).

One of the main ways that cells detect increasing mechanical force is via the Hippo-signalling pathway, discussed at length, earlier in this review. The Hippo-signalling pathway involves transduction of mechanical signals via actin across the cell cytoplasm resulting in transcriptional activation of genes via YAP/TAZ activation of the DNA binding protein TEAD [140]. Interestingly, a consensus TEAD binding sequence exists in the SDC1 promoter but not in SDC2 or SDC4 (ACATTCCA)and TEAD indeed has been shown to regulate SDC1 expression [207].

Given the role of SDC1 in maintaining MD, our data suggests a positive feedback cycle at play, where mechanical force itself may maintain PG (SDC1) expression to facilitate collagen alignment, and thus stiffness. High MD is associated with an increased risk of breast cancer development, and certainly more invasive cancers develop in dense breasts [208]. Our data suggest that mechanical force may be the main driver of this risk.

But what cellular and regulatory processes are at play? In solid malignant states, aligned collagen is a feature of the tumour/stromal interface, enabling tumour cells to move along the fibrils and thus acquire local invasive properties [209]. Given that aligned collagen potentiates a stiffened environment [210], this can directly influence cellular adherence patterns. Modelling studies supported by additional biological studies have confirmed that in soft matrices, cells favour cell–cell adhesions, where E-cadherin and β-catenin remain bound at the cell membrane, whereas higher stiff environments i.e. due to high ECM density cells favour ECM adherence, a change which results in the formation of focal contacts and translocation of β-catenin to the nucleus, engaging pro-EMT transcriptional pathways [97, 211].

Given these findings, in the context of high MD, which is an environment of increased mechanical force on epithelia, a loosening of cell–cell and strengthening of cell-ECM may result in local invasion leading to metastatic growth facilitated by EMT.

## Take the Pressure Down: Ways to Reduce the Detrimental Effects of Force within Tissues

If increased and persistent mechanical force on cells drives a self-perpetuating cycle of ECM production and subsequent increased force, and this is associated with cancer development, perhaps cancer prevention strategies should be targeted to intercept this cycle. Certainly in idiopathic pulmonary fibrosis, which is associated with the development of lung cancer [212], anti-fibrotic agents are protective [213]. Furthermore, the abundance of fibroblast activation protein (FAP), found to be more abundant in tissues with high MD [45], predicts a poor clinical response in various cancers, and is proving a viable target for inhibition to limit cancer progression [214]. This suggests that inactivating the cell that produces ECM and thus intra-tumoral pressure, the fibroblast, is a useful strategy. Cirrhosis of the liver results in high mechanical forces placed upon viable hepatocytes, turning a soft organ into a palpable, hardened mass. In this setting, anti-fibrotic agents have proven to be effective in reducing the emergence of liver cancer [215]. Indeed, tamoxifen is an effective drug to prevent breast cancer recurrence, mainly through blocking the pro-malignant effects of estrogen, however MD is reduced as well, similar in accordance with breast cancer risk reduction [50]. Although approved for preventative use in high breast cancer risk women, uptake rates are very low, due in part to the side effect profile, which can be intolerable in some women [216]. In the breast however, is it best to degrade the source of the force (break down the stromal tissue) or to modify cellular responses to mechanical force? Some might argue that agents acting to break down FGT in MD may have unfavourable outcomes for women, such as loss of shape and breast orientation. Topically-delivered agents to intercept stiffness signalling, such as specific inhibition of nucleoplasmic LMNA localisation, for example, might be a preferred option.

## Conclusion

This review provides a detailed appraisal of the inter-relationships between mechanical environment of breast tissue, its extracellular matrix, and breast cancer risk. We start with an empirical overview of mechanical properties of soft tissues and experimental techniques of their characterisation. We then discuss the biophysical basis and mathematical approaches to the modelling of stiffness of the two major components of breast tissue: fibroglandular and adipose tissue. In regards to this aspect of the review, we highlight some mathematical models in areas related to tumour initiation and growth. However, it appears that we are yet to see a single model which directly explores the connections between mechanical pressure, proteoglycan expression, and MD. Mathematically this is an interesting challenge, and biologically it would provide a useful framework to test and explore hypotheses, for example in relation to changes in MD and clinical oncology [217–219]. Furthermore, combining biological experimentation with mathematical modelling can improve our mechanistic understanding of the processes and may reduce experimental costs and time.

We illustrate a breast-specific microenvironment where increased mechanical force is detrimental and leads to carcinogenesis, and contrast this with the positive role that cellular-level mechanical forces play in health. We consider prospective experimentally based studies demonstrating the induction of various intracellular signalling pathways leading to transcriptional modifications within genes.

The central theme of this review is the evidence supporting a self-perpetuating breast tissue micromechanics cycle, whereby mechanical force may in itself promote proteoglycan synthesis, driving an increase of mammographic density. Approaches based upon interrupting this positive feedback cycle of mechanical force may provide a novel avenue of cancer prevention.
